# Effects of Semelil (ANGIPARS™) on focal cerebral ischemia in male rats

**Published:** 2010

**Authors:** M Asadi-Shekaari, H Eftekhar Vaghefi, A Talakoub, HR Khorram Khorshid

**Affiliations:** 1 Neuroscience Research Center; 2 Department of Anatomy, Faculty of Medicine, Kerman Medical University of Medical Sciences, Kerman; 3 Genetic Research Center, Social Welfare and Rehabilitation Sciences University, Tehran, Iran

**Keywords:** Middle Cerebral Artery (MCAO), Re-perfusion, Hippocampus

## Abstract

**Background and the purpose of the study:**

Cerebral ischemia is one of the main causes of long term disability and death in aged populations. Many herbal drugs and extracts have been used for the treatment of cerebral ischemia induced insults. This study was designed to investigate the protective effect of Semelil (ANGIPARS™), a new herbal drug, on focal cerebral ischemia in male rats.

**Material and methods:**

Male rats were divided into five groups: sham-operated, ischemic animals treated with distilled water as vehicle, ischemic animals treated with 1, 10 and 100 mg/kg of Semilil respectively. Middle cerebral artery occlusion (MCAO) model was used in NMRI rats and neuronal injury analyzed in hippocampal CA1 sector after 48 hrs of Middle Cerebral Artery (MCAO).

**Results:**

Results of this study showed that treatment with semelil attenuated ischemic damages and has positive effects on focal cerebral ischemia.

## INTRODUCTION

In spite of many efforts, cerebral ischemia or stroke still represents the third leading cause of death and the most important source of long-term disability in the world. Many herbal drugs including *Nigella sativa L.* extracts (1); green tea extract (2) and date fruit extract (3) have been used for the treatment of this disease.

*Melilotus officinalis* has been introduced as a component of a new drug by trade name of Semilil (ANGIPARS™). In vivo studies in rodents and dogs and also in vitro studies in some established cell lines have approved its safety (4–6). Previous studies have shown beneficial effects of Semilil such as improvement of blood circulation, reduction of inflammation, improvement of lymphedema, and immune system (7–10). Results of clinical trials on Semilil indicated its safety and efficacy in human diabetic foot ulcer (11–13). This drug has been found to have strong antioxidant components such as 7 hydroxy coumarin, flavonoids, and oleanene glucuronide (9, 14). Since some of these properties (anti-inflammatory and antioxidant) may be useful in the treatment of the damages that inevitably followed by cerebral ischemia, in this study the protective effects of Semilil on focal cerebral ischemia in male rats was investigated.

## MATERIALS AND METHODS

Semelil herbal extract (ANGIPARS™) was generously prepared and delivered by ParsRoos Co. (Tehran, Iran).

NMRI male rats weighing 220–280 g were used in accordance with Kerman Neuroscience Research Center (EC/KNRC/88-6) legislation on the use and care of laboratory animals. Animals were clinically normal, free of obvious infection or inflammation. The animals were divided into 5 different groups: sham-operated group (n = 4), ischemic animals treated with distilled water as vehicle (Ischemia + Vehicle) (n = 5), ischemic animals treated with 1, 10 and 100 mg/kg of Semilil (Ischemia + 1 mg/kg of Semilil, n = 4; Ischemia + 10 mg/kg of Semilil, n = 4; Ischemia + 100 mg/kg of Semilil, n = 31). Semilil was injected intraperitoneally 30 min after induction of ischemia.

### 

#### Transient focal cerebral ischemia

The MCAO was induced by using an intraluminal monofilament model (15). In brief, the animals were anesthetized with chloral hydrate (Merck, 400 mg/kg), placed in supine position on a heated pad, with body temperature maintained at 37±0.5°C using rectal thermometer. Under the operating microscope, the right common carotid artery, external and internal carotid arteries were exposed. After blocking all branches of the external carotid artery and extra cranial branches of the internal carotid artery, a 3-0 nylon intraluminal filament was introduced into the cervical internal carotid artery and advanced intracranially to block blood flow into the Middle Cerebral Artery (MCA). After 30 min, the intraluminal filament was withdrawn and blood flow resumed (Ischemia/ Reperfusion) (I/R). After full recovery, neurological evaluation was performed to ensure occurrence of MCAO, and animals without clinical signs were then excluded from the experiment.

#### Histological assessments

**Figure 1:**
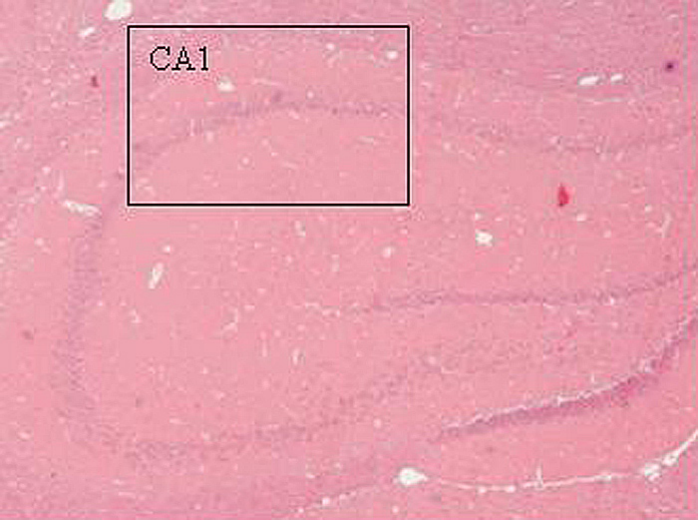
Photomicrograph of coronal section of rat hippocampus. The box indicates the area which was analyzed in the study.

The animals were anesthetized with chloral hydrate (Merck) (400 mg/kg BW, intraperitoneally) and killed by cardiac perfusion of saline followed by 10% formaldehyde in 0.1 M sodium phosphate-buffered solution (PBS, pH of 7.5). Subsequent to perfusion, the brains were carefully removed and stored in the perfusion fixation at 4°C for a minimum of 24 hrs. The brains were sectioned coronally (5 µm) by using a microtome set. Brain sections were subjected to H&E and immunhistochemistry (IHC) by using TUNEL staining. Neuronal damage was calculated based on the number of degenerated neurons to that of both surviving and degenerated neurons in 3 separate areas of the CA1 sector at a magnification of X400 (medial, middle and lateral parts, Fig 1) (16).

#### TUNEL staining

The terminal deoxynucleotidyl transferase (TdT)-mediated in situ dUTP nick end-labeling (TUNEL) assay was utilized on the brain sections using the cell death detection Kit POD (Roche; Indianapolis, IN). A dark brown color indicating DNA breaks developed after incubation with DAB (3-3́-diamonobenzidine tetrachloride) and hydrogen peroxide. The method was used to verify the cell death in hippocampal CA1 sector (17).

#### Statistical analyses

The data were presented as mean±SEM. One way ANOVA and Tukey-Kramer multiple post hoc test was used to compare data between different groups and p < 0.05 was considered statistically significant difference.

## RESUlTS

**Figure 2:**
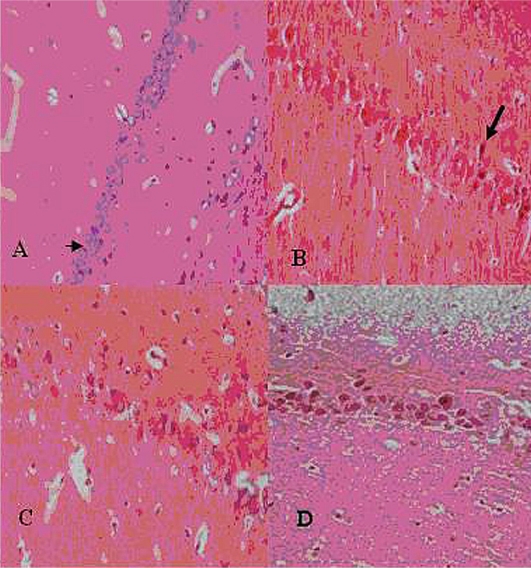
Protective effects of Semelil (ANGIPARS™) in the CA1 hippocampal neurons of rats after transient cerebral ischemia. Histopathological changes in CA1 area of hippocampus: (A) sham-operated (small arrow show the surviving neuron), (B) vehicle (large arrow shows degenerated neuron), (C) 1mg/kg of Semelil treated group, and (D) 10mg/kg of Semelil treated group. Magnification × 400. H&E staining.

Light microscopic evaluations showed morphological changes in the CA1 sector after 48 hrs of MCAO. Most of pyramidal neurons of the area showed marked injury due to 30 min ischemia and 48 hrs reperfusion. In the sham group, the morphology of neurons in CA1 sector was normal. Meanwhile, most of the neurons in ischemic group showed degenerative changes including: extensively dark piknotic nuclei and shrunken cytoplasm. In experimental groups, the severity of degenerative changes in nucleus and cytoplasm was lesser than that in ischemic groups (Fig 2).

Neuronal counting showed significant difference between ischemic + 1 mg/kg of Semilil (p < 0.05) and ischemic + 10 mg/kg of Semilil (p < 0.05) groups compared to vehicle group.

Due to the high mortality rate of the animals in Ischemia + 100 mg/kg of Semilil group, neuronal counting was not performed for this group.

**Figure 3:**
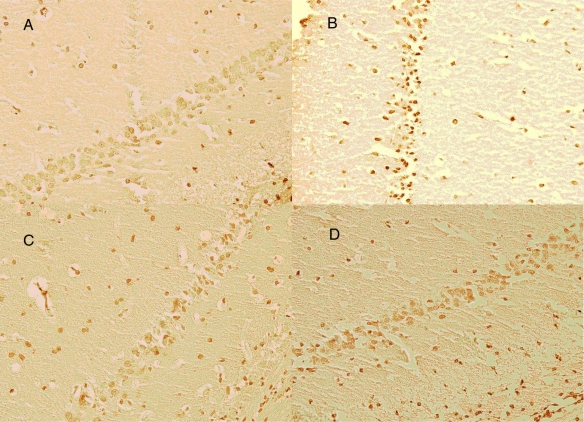
Immunohistochemical analysis of TUNEL in CA1 area of rat hippocampus for control and sham-operated (A), Ischemic (B), treated group with 1 mg/kg of Semelil (C) and treated group with 10 mg/kg of Semelil (D). Arrow show the apoptotic cells. Magnification × 400. TUNEL staining.

Neuronal injury in the CA1 area following ischemia/ reperfusion was also examined by TUNEL method. There were no TUNEL positive (+) cells in the CA1 area in the sham-operated group but many TUNEL posetive cells were detected in the vehicle group (Fig 3).

**Figure 4:**
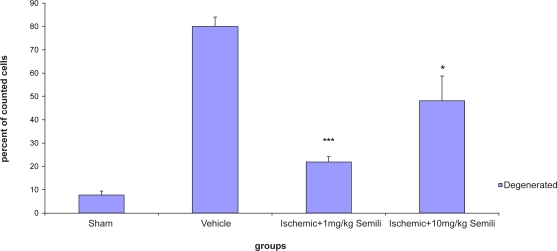
Effect of Semelil on hippocampal CA1 cell death induced by 30 min MCAO followed by 48 hrs reperfusion. Results are expressed as mean±S.E and data were analyzed by One-way ANOVA followed by Tukey-Kramer multiple comparisons test. ***Significantly different from control.

## DISCUSSION

To our knowledge, this is the first study that provided evidence of effectiveness of Semilil in cerebral ischemia in a rat I/R model.

**Table 1:** Effect of Semelil on hippocampal CA1 neuronal death in Middle Cerebral Artery Occlusion rats.

GROUP	(ALL)*	(DC)**	(DC/ALL×100)***
Sham-operated	328.75	25	7.68
Ischemic + Vehicle	304.75	245	79.97
Ischemic + 1mg/kg Semelil	340.33	74.66	21.87#
Ischemic + 10mg/kg Semelil	328.00	151.5	48.06#

Six group (n = 6) were counted.

* Average numbers of all of neurons

** Average numbers of the degenerated cells (DC)

*** Percent average of the cells death (DC/ All×100)

# P < 0.05 for Semelil (1 and 10 mg/kg)

selenium is a new drug product containing herbal extract with known beneficial effects especially on diabetic foot. Some of its components are urea, selenium, fructose and *Melilotus officinalis* extract as declared by manufacturer. Previous studies have shown that urea could improve cerebral blood flow and oxygenation and it may work the same as manitol, a known neuroprotective (18). Selenium is a known potent antioxidant agent that may have neuroprotective activity (19). *Melilotus officinalis* extract could reduce activation of circulating phagocytes and it has anti inflammatory, antiedematous and antioxidants effects. (8, 9, 14). On the other hand, experimental evidences have shown that fructose can induce inflammatory response that may worsen the ischemic damage (20).

Brain is almost absolutely dependent on the continuous flow of oxygen and glucose to undergo oxidative phosphorylation for energy production. The first result of cerebral blood flow reduction is decline of substrates, mainly oxygen and glucose that causes accumulation of lactate via anaerobic glycolysis. Acidosis may augment free radical formation, interfering with intracellular protein synthesis and worsen ischemic brain damages (21). In addition, re-perfusion in the brain after ischemia induces an inflammatory response that may exacerbate initial levels of tissue damage. There are a number of possible mechanisms by which post-ischemic inflammation could contribute to damages, including production of toxic mediators such as NO by activated inflammatory cells and vascular occlusion by neutrophils.

It has been reported that inhibition of reactive oxygen species generation, inflammatory cell activation, pro-inflammatory cytokine production, apoptotic gene induction provides neuroprotective effects against cerebral I/R injury (22).

Semilil has many components with neuroprotective properties that may explain the observed effects. At the present time, the exact protective mechanism of Semilil on cerebral ischemia is not known. This study was just concentrated on general effect of Semilil on neuronal survival in hippocampus and not its mechanism. Further studies focusing on microcirculation of prepost reperfusion and inflammatory factors are required to find the related mechanisms.
